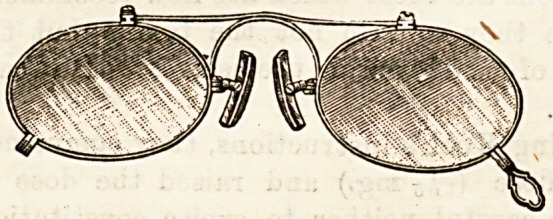# New Appliances and Things Medical

**Published:** 1897-11-20

**Authors:** 


### NEW APPLIANCES AND THINGS MEDICAL.

[We shall be glad to receive, at our Office, 28 & 29, Southampton Street, Strand, London, W.C., from the manufacturers, specimens of all new preparations and appliances which may be brought out from time to time.]

### THE REVLUC PINCE-NEZ.

(George Culver, White Lion Street, Pentonville, N.)

This is a peculiarly ingenious form of pince-nez. The frames holding the glasses are connected by a strong horizontal spring which, as in other forms of pince-nez, passes over the nose. This allows the lenses to be separated to a certain extent from one another. The ingenuity of the arrangement, however, lies in this, that a very small movement of the lenses produces a very wide opening of the cork-covered plaquets by which the pince-nez is held in place upon the nose. It is difficult to explain the exact mechanism without a diagram, but it may be stated generally that the plaquets are fixed on the glasses by a hinge in such a manner that, while the glasses move parallel with the face, the cork surfaces which are to hold the nose open towards the face, and thus grip the nose in a manner quite different from that which is found in those forms in which the plaquets are attached to and move with the lenses. The Revluc is light and elegant, and is certainly both comfortable and efficient.

It enables quite heavy glasses to be worn, and from the very minute amount of alteration in the distance of the lenses from centre to centre, produced by the necessary adjustment of the plaquets to the nose, it is quite possible, when once the proper position has been obtained, to use the Revluc for cylindrical lenses. Altogether this is a great advance on many of the common forms of pince-nez.

## Figures and Tables

**Figure f1:**
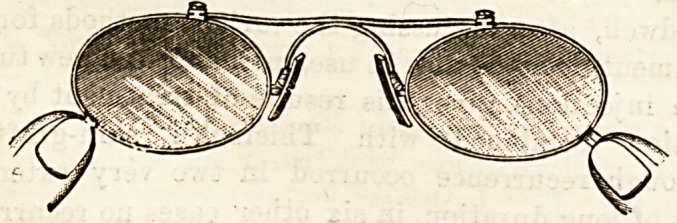


**Figure f2:**